# Prenatal diagnostic outcomes and positive predictive values in 1,326 pregnancies with high-risk non-invasive prenatal testing results

**DOI:** 10.3389/fgene.2026.1899719

**Published:** 2026-10-27

**Authors:** Runxuan Du, Bingyi Zhao, Xuedong Shi, Yingjie Gao, Yali Li, Bo Qiu

**Affiliations:** 1 Department of Reproductive Genetics, Hebei General Hospital, Shijiazhuang, China; 2 Hebei Clinical Research Center for Birth Defects, Shijiazhuang, China; 3 Department of Pharmacy, Hebei General Hospital, Shijiazhuang, China

**Keywords:** chromosomal aneuploidy, genetic counseling, non-invasive prenatal testing, positive predictive value, prenatal diagnosis, sex chromosome abnormality

## Abstract

**Objective:**

To investigate the prenatal diagnostic outcomes of pregnant women with high-risk non-invasive prenatal testing (NIPT) results and to explore factors influencing the positive predictive value (PPV) of NIPT.

**Methods:**

Clinical data from 1,326 pregnant women with high-risk NIPT results who underwent invasive prenatal diagnosis at Hebei General Hospital between January 2014 and December 2023 were retrospectively analyzed. Chromosomal karyotyping, fluorescence *in situ* hybridization (FISH), and/or copy number variation sequencing (CNV-seq) were used as confirmatory diagnostic methods. PPVs were compared across different types of chromosomal abnormalities, maternal age groups, Z-score categories, ultrasound findings, and before and after implementation of the free NIPT screening policy.

**Results:**

Among the 1,326 pregnancies with high-risk NIPT results, 648 were confirmed as true positives, yielding an overall PPV of 48.87%. The PPVs for trisomy 21 (T21), trisomy 18 (T18), trisomy 13 (T13), and sex chromosome abnormalities (SCAs) were 78.61%, 56.36%, 10.53%, and 47.11%, respectively. The PPVs for common autosomal trisomies were higher in the advanced maternal age group than in the non-advanced maternal age group. After implementation of the free screening program, the PPVs for T21, T18, and T13 showed a slight numerical increase, the difference between groups was not statistically significant. PPVs increased with higher Z-scores and were further elevated when multiple ultrasound soft markers or structural anomalies were present. Among SCAs, the PPV for sex chromosome gains was significantly higher than that for sex chromosome losses.

**Conclusion:**

NIPT demonstrated high screening performance for T21 and T18, whereas T13 and SCAs were associated with relatively high false-positive risks. Clinical interpretation of high-risk NIPT results should integrate maternal age, Z-score, ultrasound phenotype, and confirmatory prenatal diagnostic findings to facilitate stratified genetic counseling and optimize pregnancy management.

## 1 Introduction

Chromosomal aneuploidy is an important genetic cause of adverse pregnancy outcomes. Among these abnormalities, trisomy 21 (T21), trisomy 18 (T18), and trisomy 13 (T13) are the most frequently encountered in clinical practice. Conventional maternal serum screening has relatively low sensitivity and a high false-positive rate. With the discovery of cell-free fetal DNA (cffDNA) in maternal peripheral blood, high-throughput sequencing-based non-invasive prenatal testing (NIPT) has been widely adopted in clinical practice, substantially improving the screening performance for common autosomal aneuploidies (Lo et al., 1997; Bianchi and Chiu, 2018).

Although NIPT has high sensitivity and specificity for the three common autosomal trisomies, cffDNA is mainly derived from apoptotic placental trophoblasts. Therefore, NIPT remains a screening tool rather than a diagnostic test. In clinical practice, NIPT results may be affected by multiple factors, including confined placental mosaicism (CPM), maternal chromosomal abnormalities, occult maternal malignancy, vanishing twin, and low fetal fraction. These factors may lead to variation in the positive predictive value (PPV) across different types of chromosomal abnormalities (Liehr, 2022). Therefore, a high-risk NIPT result should not be equated with a definitive diagnosis. Evaluating the PPV of NIPT in real-world clinical practice is essential for guiding subsequent clinical decision-making.

In this study, we retrospectively analyzed the invasive prenatal diagnostic outcomes of 1,326 pregnant women with high-risk NIPT results who were managed at our hospital between 2014 and 2023. We further investigated the influence of chromosomal abnormality type, maternal age stratification, and ultrasound phenotype on the PPV of NIPT, aiming to provide theoretical evidence and clinical reference for genetic counseling in pregnancies with high-risk NIPT findings.

## 2 Methods

### 2.1 Study population

Pregnant women who presented to Hebei General Hospital between January 2014, and December 2023, because of high-risk NIPT results and subsequently underwent invasive prenatal diagnosis were enrolled in this retrospective study. The provincial government-funded free first-tier NIPT screening program commenced in July 2019, which separated our cohort into pre-program and post-program groups with different testing conditions. During the study period (January 2014–December 2023), 1,364 singleton pregnant women with high-risk NIPT results attended genetic counseling. Among them, 1,326 (97.21%) underwent invasive prenatal diagnosis and constituted the final study cohort. 38 patients (2.79%) were excluded: 15 declined invasive testing after comprehensive counseling, 14 suffered spontaneous miscarriage or intrauterine fetal death before sampling, and 9 were lost to follow-up or transferred to other hospitals.

The inclusion criteria were as follows.

1. Singleton pregnancy or pregnancy managed clinically as a singleton pregnancy;
2. High-risk result on NIPT;
3. Invasive prenatal diagnosis performed at our hospital;
4. Complete clinical and laboratory data.

The exclusion criteria were as follows.

1. High-risk result detected by expanded NIPT (NIPT-plus);
2. Twin or multiple pregnancy;
3. Missing key clinical data or loss to follow-up;
4. Women who did not undergo invasive prenatal diagnosis after genetic counseling.

### 2.2 NIPT procedure

NIPT was performed by different hospitals or third-party clinical laboratories. Post-implementation of the free NIPT screening program (565 cases). All samples were centrally tested by BGI via standardized shallow whole-genome sequencing. EDTA-anticoagulated maternal blood was transported to laboratories within 96 h for cffDNA extraction, library construction and bioinformatic analysis following BGI protocols. Sequencing was performed on the MGISEQ-2000 platform with 35 bp single-end reads and a fixed yield of 6 million raw reads per sample. Chromosomal Z-scores were calculated, and autosomal aneuploidies were classified high-risk at |Z| ≥ 3. Testing required a minimum fetal DNA fraction of 4%. Pre-implementation of the free NIPT screening program (761 cases). All samples underwent shallow whole-genome sequencing using three mainstream platforms: Illumina (199 cases), Ion Torrent (324 cases), and BGI (214 cases). 24 archived samples lacked traceable technical metadata. All certified laboratories followed national NIPT guidelines.

### 2.3 Invasive prenatal diagnosis

All pregnant women with high-risk NIPT results underwent invasive prenatal diagnosis at the Department of Reproductive Genetics, Hebei General Hospital, after providing written informed consent. Amniocentesis or cordocentesis was performed according to gestational age, placental location, amniotic fluid volume, and clinical indications.

After sample collection, conventional G-banded chromosomal karyotyping was performed. FISH or CNV-seq was additionally conducted when necessary to further clarify chromosomal numerical abnormalities, structural abnormalities, or mosaicism. Prenatal diagnostic results were used as the reference standard for determining whether NIPT results were true positive or false positive.

### 2.4 Observational indicators

Clinical baseline characteristics, NIPT screening reports, and invasive prenatal diagnostic results were collected and analyzed. Chromosomal karyotyping, FISH, and/or CNV-seq results were used as the diagnostic reference standard. The PPVs of different types of high-risk NIPT findings, including T21, T18, T13, SCAs, microdeletion/microduplication syndromes (MMS), rare autosomal trisomies (RATs), and combined chromosomal abnormalities, were calculated and compared. The PPV was calculated as follows: PPV = number of true-positive cases/ number of high-risk NIPT cases.

NIPT Z-scores were stratified to analyze the relationship between different Z-score intervals and the PPV of each type of chromosomal abnormality. Maternal age, ultrasound soft markers, and structural anomalies were also evaluated to explore the effects of age stratification and ultrasound phenotypes on the PPV of NIPT.

Pregnancy outcomes of confirmed positive cases were followed, including continuation of pregnancy, termination of pregnancy, and reasons for induction of labor. Particular attention was paid to clinical decision-making characteristics and associated factors after confirmed diagnosis of SCAs. In addition, using implementation of the free NIPT screening policy as a time point, we compared the PPVs for common trisomies before and after policy implementation to evaluate the effect of a standardized screening model on testing performance.

### 2.5 Statistical analysis

Statistical analyses were performed using SPSS version 26.0. Categorical variables were expressed as numbers and percentages. Between-group comparisons were conducted using the chi-square test or Fisher’s exact test, as appropriate. A two-sided *P* value < 0.05 was considered statistically significant.

## 3 Results

### 3.1 Prenatal diagnostic outcomes and PPVs of high-risk NIPT results

A total of 1,326 pregnant women with high-risk NIPT results were included in this study. Among them, 648 cases were confirmed as true positives by prenatal diagnosis, resulting in an overall PPV of 48.87%.

The PPVs for T21, T18, and T13 were 78.61% (294/374), 56.36% (62/110), and 10.53% (8/76), respectively. The PPV for SCAs was 47.11% (212/450). The PPVs for MMS, RATs, and combined chromosomal abnormalities involving two chromosomes were 37.65% (61/162), 7.41% (10/135), and 5.26% (1/19), respectively (Table 1).

**TABLE 1:** Prenatal diagnostic outcomes and positive predictive values according to different types of high-risk NIPT results.

Type of high-risk NIPT result	Number of high-risk NIPT cases	True-positive cases	False-positive cases*	PPV (%)
T21	374	294	80	78.61
T18	110	62	48	56.36
T13	76	8	68	10.53
SCA	450	212	238	47.11
MMS	162	61	101	37.65
RAT	135	10	125	7.41
Two chromosomal abnormalities	19	1	18	5.26
Total	1,326	648	678	48.87

PPV, positive predictive value; T, trisomy; SCA, sex chromosome abnormality; MMS, microdeletion/microduplication syndrome; RAT, rare autosomal trisomy.

* False-positive cases included cases with negative prenatal diagnostic results or prenatal diagnostic results inconsistent with the NIPT, finding.

### 3.2 Comparison of PPVs of high-risk NIPT results between different maternal age groups

Pregnant women were divided into a non-advanced maternal age group (<35 years) and an advanced maternal age group (≥35 years) according to the expected date-of-delivery age. The PPV for T21 was highest in the advanced maternal age group, reaching 88.05%, which was significantly higher than that in the non-advanced maternal age group (71.63%). The PPV for T18 was also significantly higher in the advanced maternal age group than in the non-advanced maternal age group (70.21% vs. 46.03%). No statistically significant differences were observed between the two age groups for T13, SCA, MMS, or RAT (Table 2).

**TABLE 2:** Comparison of positive predictive values of high-risk NIPT results between different maternal age groups.

Type of high-risk NIPT result	PPV in women <35 years (%)	PPV in women ≥35 years (%)	χ^2^ value	*P* value
T21	71.63 (154/215)	88.05 (140/159)	14.660	<0.001
T18	46.03 (29/63)	70.21 (33/47)	6.400	0.011
T13	7.84 (4/51)	16.00 (4/25)	1.195	0.276
SCA	45.73 (166/363)	52.87 (46/87)	1.437	0.231
MMS	37.01 (47/127)	40.00 (14/35)	0.105	0.746
RAT	8.51 (8/94)	4.88 (2/41)	0.549	0.459
Total	44.69 (408/913)	60.66 (239/394)	28.088	<0.001

### 3.3 Comparison of PPVs before and after implementation of the free NIPT screening policy

We further compared the performance of NIPT before and after implementation of the free screening policy. PPVs of T21, T18 and T13 were numerically higher in samples tested after the implementation of the free screening program. Specifically, the PPVs for T21, T18, and T13 increased from 77.61%, 55.56%, and 8.70% before program implementation to 81.13%, 57.89%, and 13.33% after program implementation, respectively. However, these differences were not statistically significant (Table 3).

**TABLE 3:** Comparison of positive predictive values of high-risk NIPT results before and after implementation of the free screening policy.

Type of high-risk NIPT result	PPV after free screening policy (%)	PPV before free screening policy (%)	χ^2^ value	*P* value
T21	81.13 (86/106)	77.61 (208/268)	0.560	0.454
T18	57.89 (22/38)	55.56 (40/72)	0.055	0.814
T13	13.33 (4/30)	8.70 (4/46)	0.415	0.520

### 3.4 Prenatal diagnostic results and pregnancy outcomes in high-risk SCA cases

Among the 450 high-risk SCA cases, the overall PPV was 47.11%. The PPV for sex chromosome gains was significantly higher than that for sex chromosome losses (73.01% vs. 20.98%; Table 4).

**TABLE 4:** Prenatal diagnostic results in high-risk sex chromosome abnormality cases.

Type of high-risk SCA result	Number of high-risk NIPT cases	True-positive cases	PPV (%)	χ2 value	P value
Sex chromosome loss	224	47	20.98	122.211	<0.001
Sex chromosome gain	226	165	73.01		
Total	450	212	47.11	​	​

χ^2^ and P value represent the comparison of PPV, between sex chromosome loss and sex chromosome gain groups.

Among confirmed SCA cases, the overall pregnancy termination rate was 76.89%. Termination rates differed markedly by karyotype: the highest rate was observed for 47, XXY (91.36%), followed by 47, XYY (79.17%) and 45, X or mosaicism (67.31%), whereas 47, XXX had the lowest termination rate (51.61%; Table 5).

**TABLE 5:** Pregnancy outcomes of confirmed sex chromosome abnormality cases.

Type of sex chromosome abnormality	Number of confirmed cases	Number of pregnancy terminations	Termination rate (%)
45, X and mosaicism	52	35	67.31
47, XXX	31	16	51.61
47, XXY	81	74	91.36
47, XYY	48	38	79.17
Total	212	163	76.89

### 3.5 Relationship between Z-score and PPV in high-risk common trisomy cases

We further analyzed the association between Z-score stratification and PPVs for common trisomies (Table 6). The results showed that the PPVs for T21 and T18 increased with higher Z-scores. For T21, when the Z-score was in the borderline range of 3 to < 4, the PPV was 25.00%; when the Z-score was ≥ 6, the PPV increased to 85.19%; and when the Z-score was ≥ 13, the PPV reached 100.00%. T18 showed a similar trend of increasing PPV with increasing Z-score. In contrast, no clear linear association was observed between Z-score and PPV for T13.

**TABLE 6:** Positive predictive values of common trisomies stratified by Z-score interval.

Z-score interval	PPV for T21 (%)	PPV for T18 (%)	PPV for T13 (%)
3–<4	25.00	15.79	13.33
4–<5	48.15	38.46	0
5–<6	50.00	55.56	11.11
6–<7	85.19	60.00	0
7–<8	81.58	60.00	0
8–<9	82.61	57.14	12.50
9–<10	86.36	71.43	0
10–<11	88.24	100.00	0
11–<12	94.12	87.50	0
12–<13	95.00	83.33	0
13–<14	100.00	100.00	0
14–<15	100.00	75.00	0
≥15	95.29	71.43	50.00

### 3.6 Association between ultrasound findings and prenatal diagnostic outcomes in high-risk NIPT cases

The PPVs of different high-risk NIPT findings stratified by ultrasound manifestations are shown in Table 7. The PPVs for common trisomies (T21, T18, and T13) increased with the complexity of ultrasound abnormalities. For high-risk T21 and T18 cases, the PPV reached 100.00% when ≥ 3 ultrasound soft markers or fetal structural anomalies were present. For T13, although the overall PPV was relatively low, the PPV increased from 2.04% in cases with normal ultrasound findings to 50.00% in cases with two ultrasound soft markers.

**TABLE 7:** Positive predictive values of different high-risk NIPT results according to ultrasound findings.

Type of high-risk NIPT result	Normal ultrasound PPV (%)	Single soft marker PPV (%)	Two soft markers PPV (%)	≥3 soft markers PPV (%)	Structural anomaly PPV (%)
T21	66.67	80.00	93.55	100.00	100.00
T18	15.38	54.84	88.89	100.00	100.00
T13	2.04	9.52	50.00	—	—
SCA	48.56	42.86	35.00	100.00	75.00
MMS	40.43	29.17	40.00	0	33.33
RAT	6.82	8.33	0	—	25.00

By contrast, the PPV of SCA did not show a clear increasing trend with the number of ultrasound soft markers. Nevertheless, when ≥ 3 soft markers or structural anomalies were present, the PPV remained relatively high, reaching 100.00% and 75.00%, respectively.

## 4 Discussion

In this retrospective analysis of 1,326 pregnancies with high-risk NIPT results, the overall PPV was 48.87%. The PPVs for T21, T18, T13, and SCAs were 78.61%, 56.36%, 10.53%, and 47.11%, respectively. These findings are broadly consistent with recent domestic and international multicenter cohort studies (Yang et al., 2025; Zhou et al., 2025), supporting the robust performance of NIPT in screening for common autosomal aneuploidies.

However, the PPV for T13 remained relatively low. This may be partly explained by the low GC content of chromosome 13, which can introduce sequencing bias during next-generation sequencing library preparation and amplification, thereby affecting the stability of bioinformatic algorithms. In addition, fetuses with T13 are often associated with placental dysplasia, which may reduce the amount of cffDNA released into maternal circulation and make the fetal signal more susceptible to background noise (Xue et al., 2019). To address these limitations, future studies may improve bioinformatic performance by incorporating optimized GC-correction models and combining NIPT with first-trimester ultrasound structural screening, thereby reducing the false-positive rate for T13 (Dai et al., 2025).

Our results also showed that the PPVs for common trisomies were numerically higher after implementation of the government-funded free screening program, although these differences did not reach statistical significance. This trend may be attributable to the enhanced standardization of laboratory quality control systems under the public health program, including more consistent sequencing platforms, optimized bioinformatic algorithms, and standardized sample transportation and processing procedures. However, the lack of statistical significance may be explained by several factors. First, small subgroup sizes limited statistical power, especially for T18 and T13. Second, the policy expanded NIPT access to a broader population, including lower-risk women, which could partially offset improvements in laboratory performance. Third, biological variability in factors such as maternal age distribution and fetal fraction across time periods may have influenced the results. Nevertheless, a closed-loop quality control system integrating clinical practice and laboratory workflow remains essential for ensuring and continuously improving the performance of NIPT. A large real-world cohort from the Hebei free NIPT program, enrolling roughly 1.19 million pregnancies, reported PPVs of 81.76%, 63.73% and 17.3%for T21, T18 and T13 respectively (Liu et al., 2026), further supporting the clinical utility of this public health initiative.

The Z-score stratification analysis showed a positive association between Z-score and PPV, particularly for T21 and T18, consistent with previous studies (Chen et al., 2022; Tang et al., 2024). Borderline low Z-scores are more likely to be affected by complex maternal–fetal biological factors, especially confined placental mosaicism, which is a major contributor to false-positive NIPT results (Pan et al., 2025). Evaluating the clinical significance of Z-scores can help implement stratified genetic counseling. For pregnant women with extremely high Z-scores for T21 or T18, timely invasive prenatal diagnosis should be recommended. For those with low Z-scores, particularly in cases of high-risk T13 or advanced maternal age, clinicians should carefully explain potential causes of false-positive results to reduce psychological distress triggered by a “high-risk” report (Eggenhuizen et al., 2024; Rosenblum et al., 2024).

Consistent with previous reports, the overall PPV for SCAs in this study was 47.11%, and the PPV for sex chromosome gains was significantly higher than that for sex chromosome losses (Luo et al., 2021; Zhen et al., 2025). The high false-positive rate for 45, X may be mainly attributable to biological and technical factors, including fluctuations in the GC content of the X chromosome and maternal sex chromosome mosaicism (Chang et al., 2025; Lin et al., 2025). After birth, individuals with SCAs may present with reproductive developmental abnormalities or mild cognitive impairment, some of which may be improved through clinical intervention (Legue et al., 2025). A recent study further showed that infants prenatally diagnosed with sex chromosome trisomies had significantly increased risks of breastfeeding difficulties, positional torticollis, eczema, food allergy, cardiac septal defects, and renal structural abnormalities during the first year of life (Tartaglia et al., 2025). Therefore, for high-risk SCA cases, a multidisciplinary team (MDT) approach should be adopted to provide evidence-based prognostic counseling while fully respecting maternal informed choice.

Because common fetal aneuploidies are frequently accompanied by characteristic ultrasound findings, prenatal ultrasound and NIPT have strong complementary value in clinical evaluation (Yuan et al., 2023; Andonotopo et al., 2025; Bianchi et al., 2025). In the present study, the combined “imaging plus molecular genetics” strategy substantially improved the detection efficiency of chromosomal abnormalities. When high-risk NIPT results were accompanied by fetal structural anomalies or ≥ 3 ultrasound soft markers, the PPVs for T21 and T18 reached 100.00%. Moreover, for T13 and SCAs, which generally have lower overall PPVs, the presence of ultrasound abnormalities also significantly increased their predictive value.

These findings highlight the critical role of prenatal ultrasound in refining NIPT predictive performance, yet clinical practice faces practical limitations related to inconsistent timing of different prenatal tests and resulting maternal psychological distress. NIPT can be performed from gestational week 10, amniocentesis is available at week 16, and complete fetal structural ultrasound is routinely arranged near week 20. This prolonged waiting interval for confirmatory diagnosis commonly induces notable anxiety among women with high-risk NIPT findings. As reported by Scott et al. (2023), late first-trimester ultrasound substantially modifies NIPT PPV for most chromosomal abnormalities and alleviates patients’ anxiety during this waiting period. Improved ultrasound technology enables early identification of fetal soft markers, making sonographic evaluation a necessary preliminary step prior to invasive prenatal testing. Clinicians should adopt individualized management by integrating ultrasound manifestations, NIPT Z-scores and maternal age. Pregnant women with high-risk T21 or T18 results accompanied by abnormal ultrasound markers require timely invasive diagnostic testing. For conditions with inherently low PPV including T13 and sex chromosome aneuploidies, genetic counselors need to fully communicate the elevated risk of false-positive findings. Sustained targeted genetic counseling should be delivered throughout the waiting phase to mitigate patient anxiety before final diagnostic outcomes.

In summary, NIPT has substantial clinical value as a prenatal screening tool, particularly for T21 and T18. Nevertheless, false-positive results remain relatively frequent for certain karyotypes, especially T13, 45, X, RATs, and some microdeletion/microduplication abnormalities. In addition, this study has several limitations. First, it was a single-center retrospective analysis, which may limit the generalizability of the findings. Second, approximately 2.79% of women with high-risk NIPT results were excluded due to refusal of invasive testing or miscarriage before the procedure. Given the small proportion, the impact on PPV estimates is likely minimal, though this should be considered when interpreting findings. Third, placental tissue verification was not available for false-positive cases, limiting our ability to confirm the contribution of confined placental mosaicism. Additionally, this 10-year study involved NIPT testing across multiple laboratories with varied sequencing platforms and iterative in-house bioinformatic tools. Historical samples lacked full pipeline version records. While all labs followed national NIPT standards and subgroup analyses revealed no significant PPV differences between the multi-platform pre-policy period and unified BGI platform post-policy period, minor vendor-specific technical bias cannot be fully excluded. Furthermore, fetal fraction (FF) data were unavailable for this retrospective cohort. Low FF may increase false-negative risk and alter PPV, particularly for T13 and rare autosomal trisomies. Future prospective studies with standardized FF recording are required to explore the association between FF and the relatively low PPV of these chromosomal anomalies. Future integration of third-generation sequencing technologies and artificial intelligence-based algorithms may further optimize the performance of NIPT and improve the precision prevention and control of genetic birth defects (Liu et al., 2025).

## Data Availability

The original contributions presented in the study are included in the article/supplementary material, further inquiries can be directed to the corresponding author.
